# Face processing in Williams syndrome is already atypical in infancy

**DOI:** 10.3389/fpsyg.2015.00760

**Published:** 2015-06-15

**Authors:** Dean D’Souza, Victoria Cole, Emily K. Farran, Janice H. Brown, Kate Humphreys, John Howard, Maja Rodic, Tessa M. Dekker, Hana D’Souza, Annette Karmiloff-Smith

**Affiliations:** ^1^Department of Psychological Sciences, Centre for Brain and Cognitive Development, Birkbeck, University of London, London, UK; ^2^Department of Biostatistics, Institute of Psychiatry, King’s College London, London, UK; ^3^Department of Psychology and Human Development, Institute of Education, University College London, London, UK; ^4^Department of Psychology, London South Bank University, London, UK; ^5^Institute of Child Health, University College London, London, UK; ^6^Department of Psychology, Goldsmiths, University of London, London, UK; ^7^Department of Visual Neuroscience, Institute of Ophthalmology, University College London, London, UK

**Keywords:** infancy, Williams syndrome, Down syndrome, face processing, featural, configural, nativism, progressive modularization

## Abstract

Face processing is a crucial socio-cognitive ability. Is it acquired progressively or does it constitute an innately-specified, face-processing module? The latter would be supported if some individuals with seriously impaired intelligence nonetheless showed intact face-processing abilities. Some theorists claim that Williams syndrome (WS) provides such evidence since, despite IQs in the 50s, adolescents/adults with WS score in the normal range on standardized face-processing tests. Others argue that atypical neural and cognitive processes underlie WS face-processing proficiencies. But what about infants with WS? Do they start with typical face-processing abilities, with atypicality developing later, or are atypicalities already evident in infancy? We used an infant familiarization/novelty design and compared infants with WS to typically developing controls as well as to a group of infants with Down syndrome matched on both mental and chronological age. Participants were familiarized with a schematic face, after which they saw a novel face in which either the features (eye shape) were changed or just the configuration of the original features. Configural changes were processed successfully by controls, but not by infants with WS who were only sensitive to featural changes and who showed syndrome-specific profiles different from infants with the other neurodevelopmental disorder. Our findings indicate that theorists can no longer use the case of WS to support claims that evolution has endowed the human brain with an independent face-processing module.

## Introduction

Faces provide us with important social information. We use them to guide our actions and to engage in social behavior. They are also ubiquitous in the environment. It is therefore not surprising that faces acquire a special status among visual stimuli. For instance, face recognition is more disrupted by stimulus inversion than is object recognition (Yin, 1969). There also exist adult neuropsychological patients who lose the ability to recognize objects but not faces (Moscovitch et al., 1997; Duchaine and Nakayama, 2005), and *vice versa* (Bodamer, 1990; Kanwisher, 2000; Busigny et al., 2010). Moreover, functional neuroimaging studies have revealed a region of cerebral cortex—the fusiform face area (FFA)—that is significantly more activated for faces than for non-face stimuli such as assorted objects (Kanwisher et al., 1997; McCarthy et al., 1997), strings of letters (Puce et al., 1996), animals without heads (Kanwisher et al., 1999), or the backs of human heads (Tong et al., 2000). Also, faces start to acquire their special status from a very early age. For example, neonates track moving face-like stimuli farther than other visual patterns of comparable complexity, contrast, and spatial frequency (Goren et al., 1975; Johnson et al., 1991). This, along with evidence that face processing is localized in the adult brain, has led to claims in the literature that evolution has endowed the human brain with an independent, minimally interactive, face-processing module (Kanwisher, 2000, 2010).

Further claims for this nativist, modular perspective call on a rare genetic disorder, Williams syndrome (WS: for full genotypic/phenotypic details, see Farran and Karmiloff-Smith, 2012). Adolescents and adults with WS are seriously impaired in a range of domains (e.g., spatial cognition, number, and problem solving; Donnai and Karmiloff-Smith, 2000), have an average IQ of 56 (Mervis and Bertrand, 1997), and yet they perform within the normal range on standardized face-processing tests (Bellugi et al., 1988a; Deruelle et al., 2003; Tager-Flusberg et al., 2003). This has lead to claims in the literature of an “intact,” “spared,” or “preserved” face-processing module in WS (Bellugi et al., 1988b, 1994; Wang et al., 1995). Nonetheless, the question of whether face processing is “normal” in this population or calls on atypical neuro-cognitive processes remains hotly debated (Mills et al., 2000; Deruelle et al., 2003; Tager-Flusberg et al., 2003; Karmiloff-Smith et al., 2004; D’Souza and Karmiloff-Smith, 2011).

Observational studies revealed that infants and young children with WS are fascinated with faces and spend more time looking at them than at objects (Mervis and Bertrand, 1997; Bellugi et al., 2000; Laing et al., 2002). Experimental studies also found that adolescents and adults with WS perform within (or near) the normal range on standardized face-processing tasks, such as the Benton Facial Recognition Test (Benton et al., 1983) and the Rivermead Face Memory Task (Wilson et al., 1986). But could there be different (i.e., atypical) neuro-cognitive underpinnings to their success on these tasks? For instance, rather than using normal configural processing, it is possible to recognize “faces” on the Benton test by detecting specific features within the face stimuli (e.g., a nose; Duchaine and Nakayama, 2004).

One of the classic claims in the literature about how face recognition is special and differs from object recognition is that the former relies on holistic or configural processing (Tanaka and Farah, 1993). “Holistic” processing occurs whenever a system processes the emergent features of stimuli—e.g., the overall gestalt of a face or, for instance, the area of a square, rather than the lines that make up the square (Piepers and Robbins, 2012). “Configural” information, by contrast, refers to the relationship between features and involves two levels of processing: first-order and second-order configural processing. Specifically, first-order configural information refers to the basic configuration of features (eyes above mouth), while second-order configural information refers to the brain’s computation of precise variations in the spacing between these features (see Piepers and Robbins, 2012, for discussion). An important study by Deruelle et al. (1999; see also Rossen et al., 1995) found that, relative to controls, individuals with WS are better at processing the featural than the configural information of a face. Children and adults with WS (from 7 to 23 years of age) had to decide whether two pictures of faces, presented in upright and inverted conditions, were the “same” or “different.” TD controls usually process upright faces configurally and inverted faces featurally (Young et al., 1987; Leder and Bruce, 2000), and are more successful at upright than inverted faces: known as the *face inversion effect* (Yin, 1969). By contrast, Deruelle et al. (1999) discovered that the participants with WS were less subject to the inversion effect than chronological age (CA)- and mental age (MA)-matched controls. The researchers proposed that individuals with WS have a bias to process featural over configural information, even when faces are upright. This dovetails with studies that show a similar pattern in other visuo-spatial domains in WS, leading to the claim that individuals with the disorder are “featural processors” (Pani et al., 1999; but see D’Souza et al., 2015).

However, in a later study, Deruelle et al. (2003) argued that face processing is “preserved” in individuals with WS. Children and adolescents (6–17 years) with WS were instructed to match faces to either a low- or high-spatial frequency filtered target face, with the hypothesis that low-spatial frequency filters would call upon holistic processing and high-spatial frequency filters would require configural processing. The participants with WS as well as the CA- and MA-matched controls all found it easier to process low spatially filtered faces than high ones, and did not differ significantly from each other. No effect of age was found either. It seems reasonable to conclude that all three groups had developed the ability to process faces holistically and were at ceiling (i.e., by 6 years of age).

Tager-Flusberg et al. (2003) also presented findings on face processing in WS. It was an important study because of its large sample size: 47 adolescents/adults with WS, 39 CA-matched controls. These participants were tested on a number of tasks, including the Benton and a part-whole paradigm (Tanaka and Farah, 1993). Tager-Flusberg et al. (2003) found that the surrounding face context had the same effect on individuals with WS as it did for CA-matched controls. The authors concluded that face processing is normal in WS.

However, Karmiloff-Smith et al. (2004) argued that some researchers were conflating two different concepts: “holistic” and “configural” processing, and that the findings of Deruelle, Tager-Flusberg, and others did indeed provide evidence of relatively proficient “holistic” processing in WS, but not of second-order “configural” processing which develops later in TD (Maurer et al., 2002; Mondloch et al., 2002; Liu et al., 2013). As mentioned above, first-order holistic processing occurs when a face is processed directly as a “gestalt” (i.e., fusion between different elements in an array—a low-level visual phenomena). The debate, according to Karmiloff-Smith et al. (2004) is not whether individuals with WS can process a face as a gestalt, they can, but whether they make use of featural or precise configural information (or both). And herein lies the crux of the issue, the focus of our paper: Is featural and/or configural face processing atypical in WS?

Holistic and configural face processing are both involved in normal face recognition. But they develop at different times and at different rates. Holistic processing, pace Carey and Diamond (1977), develops early in infancy (i.e., from at least 3 months of age; Turati et al., 2010), whereas configural and featural processing develops later and at a much slower rate (Liu et al., 2013). Karmiloff-Smith et al. (2004) identified both delay and deviance in face processing in adolescents and adults with WS, specifically with the processing of configural information. In other words, they found that although face processing is relatively proficient in WS, it develops atypically. This had also been confirmed by neuroimaging and event-related potential (ERP) studies of anomalous brain activation in WS during face recognition (Mills et al., 2000; Grice et al., 2003; Mobbs et al., 2004), as well as by recent developmental studies which revealed atypically developmental trajectories of configural face processing in older children with the disorder (Annaz et al., 2009; Dimitriou et al., 2014). The FFA has also been found to be larger in WS than in TD controls, which may also reflect atypical face processing (Golarai et al., 2010). Whether the WS brain starts out with a large FFA, or whether its unusual volume emerges as a result of overly focused face processing in young children (Karmiloff-Smith et al., 2012), remains an open debate. Nonetheless, these are important findings, because they highlight atypicalities in face processing in this syndrome.

In sum, there is currently no consensus on whether face processing is typical in WS. Yet evidence that face processing in WS is *prima facie* typical has been used to support the claim that evolution has endowed the human infant brain with independently functioning modules dedicated to specific functions, e.g., face processing (Kanwisher et al., 1997). So, when individuals with WS present with much more serious deficits in some domains (e.g., visuo-spatial) than others (e.g., face processing), it is taken as evidence of “impaired” and “spared” modules in WS (see D’Souza and Karmiloff-Smith, 2011, for discussion). Individuals with WS should not be seen as having a normal brain with impaired and spared parts, but rather as having a brain that is *developing* differently (Karmiloff-Smith, 1997, 1998). We hypothesize that the ability to perceive a face may appear “intact” when using basic standardized tests, but actually more sensitive measures will reveal that it develops atypically in WS. Face perception involves three different levels of processing: holistic, configural, and featural. Empirical studies (hitherto mainly of adolescents and adults) provide strong evidence—and a broad consensus—that holistic processing is relatively proficient in WS. By contrast, there is also behavioral and neural evidence from several labs (Mills et al., 2000; Deruelle et al., 2003; Grice et al., 2003; Karmiloff-Smith et al., 2004; Mobbs et al., 2004; Dimitriou et al., 2014), that configural processing may develop atypically in WS, and a possibility that featural processing is also atypical (Karmiloff-Smith, 1997). However, hitherto these processes have been examined in older children, adolescents and adults with WS. But what about infants with WS? Do they start with similar face-processing abilities to typically developing (TD) infants, with atypicality developing later, or are the atypicalities already evident in infancy? This is an important question, because even if a general consensus does emerge that face processing is atypical in older children, adolescents, and adults with WS, then we would still need to know: (1) whether there is an atypicality in featural (as well as configural) processing (see Karmiloff-Smith, 1997), (2) whether the atypicality is present early in infancy or the outcome of a protracted developmental process that has been operating under atypical constraints, and (3) whether infants with WS show the same configural processing impairment observed in adolescents and adults with WS, or whether they also show a different impairment (i.e., featural).

To answer these questions, the present study compared featural and configural processing in infants/toddlers with WS with MA-matched TD control infants/toddlers. We also included a group of infants/toddlers with Down syndrome (DS) for two reasons. First, it was important to ascertain whether the WS profile was syndrome specific or simply due to low IQ, so the two neurodevelopmental disorder groups were matched on both CA and MA. Second, DS was selected as a comparison group because there is some evidence in the literature that whereas individuals with WS show a processing bias to featural over configural information, the opposite pattern obtains for DS (Bihrle et al., 1989; Bellugi et al., 1999; but see D’Souza et al., 2015). In the current study, we presented infants with two faces, a familiar face and either a (novel) featurally-changed face or a (novel) configurally-changed one.

We hypothesized that the infants/toddlers with WS would discriminate between the familiar face and the (novel) featurally-changed face, but not between the familiar face and the (novel) configurally-changed face. We predicted that the opposite pattern would hold for DS, and that the TD controls (who usually process upright faces configurally) would display proficiency in both conditions, albeit with stronger effects in the configural condition.

## Materials and Methods

### Participants

A total of 92 infants were tested: 29 infants/toddlers with WS, 20 infants/toddlers with DS, and 43 TD controls. The children with WS or DS had been tested either for a microdeletion of the ELN gene via *fluorescence in situ hybridization* or for full trisomy 21. All participants were assessed using the Bayley Scales of Infant Development (Bayley, 1993). Data from an overall 24 infants/toddlers (eight WS, nine DS, seven TD) were excluded from the study due to fussiness or drowsiness. Table 1 shows the mean CAs and MAs for the remaining 68 participants. The groups did not significantly differ on MA, *F*_2,65_ = 0.86, *p* = 0.429 (see Results).

**Table 1:** **Mean (SD) chronological age (CA) and mental age (MA) for each group**.

**Group**	***n***	**CA in months (SD)**	**MA in months (SD)**
WS	21	26.1 (6.6)	14.0 (5.6)
DS	11	30.5 (11.7)	16.4 (6.3)
Control	36	14.3 (4.4)	14.1 (4.7)

### Stimuli and Apparatus

**FIGURE 1:**
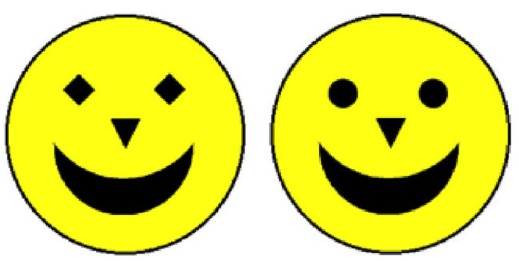
**An example of a test trial in the Featural condition.** The stimulus on the left is an example of a featurally-changed face, while the stimulus on the right is the familiarized face.

**FIGURE 2:**
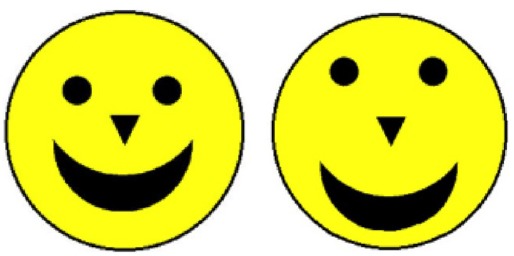
**An example of a test trial in the Configural condition.** The stimulus on the right is an example of a configurally-changed face, while the stimulus on the left is the familiarized face.

The stimuli were schematic faces: 7 cm (2.75 inch) yellow circles, with four black elements (two “eyes,” one “nose,” and one “mouth”) on a black background. This basic schematic face (Figure 1) was used as the familiarization stimulus. Two featurally-modified and two configurally-modified versions of this basic stimulus were also created. The featural changes were made by replacing the round eyes with similarly sized squares or diamonds; the configural changes were made by stretching or squashing the features toward or away from the centre by 20 pixels (see Figures 1 and 2, for examples). We opted for schematic rather than real faces for several reasons. First, it had already been shown that infants’ gaze behaviors to naturalistic faces do not differ from their behaviors to schematic faces (e.g., Farroni et al., 2005). Second, several studies have shown that the mechanisms involved in processing schematic faces are the same as those involved in processing naturalistic faces (see Johnson et al., 2015, for review). We therefore decided to use schematic faces because they are simpler to control and manipulate, can be presented in a very large format, with very strong color contrasts that capture and hold infants’ attention.

The infants were seated on their parent’s lap 60 cm (2 ft) away from a 97 cm × 56 cm (38 inch × 22 inch) monitor screen, in a dimly lit room with blank, off-white walls. The parents were instructed to look straight ahead and not at the stimuli, and to refrain from interacting with their child during the experiment. A video camera focused on the infant’s face was mounted just under the monitor. The camera was connected to a VCR and monitor screen where the experimenter, who was hidden behind curtains, could watch the infant live. For coding purposes, the experimenter used a “picture-in-picture” tool that showed the display of the infant’s screen in the corner of the experimenter’s monitor screen. The coder could therefore simultaneously see the infant’s face and the display that the infant was looking at.

### Design and Procedure

Participants were presented with eight test trials. Each test trial was preceded by four familiarization trials, except for the first test trial, which was preceded by eight familiarization trials to be sure of familiarizing the infants with the model face. The familiarization trials consisted of one yellow schematic face (the *familiarized face*) on a black background (Figure 1). The test trials consisted of two faces presented side by side—one familiarized face, and one novel face (see Figures 1 and 2, for examples). While the familiarized face remained unchanged, there were four novel faces: two *configurally-changed faces* (one with the features of the face “squashed,” the other “stretched”) and two *featurally-changed faces* (one with square eyes, one with diamond eyes). Each of these faces was presented once on the left-hand side of the screen, and once on the right. The order of the eight test trials was randomized and subsequently fixed (in the following order: featural, configural, featural, configural, featural, configural, configural, featural). So every participant was presented with the same sequence of trials. The fixed order was presented to each participant, using E-prime (Psychological Software Tools, Pittsburgh, PA, USA).

Before the start of each trial, a noisy visual distractor was used to attract the child’s attention to the screen. The trial started once the child was looking at the screen. Each familiarization trial lasted 2 s; each test trial, 4 s. The entire experiment lasted no longer than 3 min. All experimental procedures were in accordance with the Declaration of Helsinki, and were approved by the Departmental Ethics Committee, Department of Psychological Sciences, Birkbeck, University of London.

Preferential looking times were coded frame-by-frame using SuperCoder 1.5 (Hollich, 2005). The coder was blind to the experimental hypothesis. A second experimenter coded 10% of the trials. Inter-rater reliability was very high (*r* = 0.96).

## Results

### Chronological Age (CA) and Mental Age (MA) Matching

The CA data in the Control group were non-normal [*Z*_Skewness_ = 0.09, *D*(36) = 0.16, *p* = 0.019]. Because the Control data had a (continuous) uniform distribution, rather than transforming the data, a non-parametric test (Independent-Samples Kruskal–Wallis) was used. As expected, the distribution of CA was significantly different across the three groups, *H*(2) = 34.81, *p* < 0.001. However, pairwise comparisons revealed that the DS (*Mdn* = 30.00) and WS (*Mdn* = 27.10) groups did not significantly differ on CA, *U* = 3.03, χ^2^ = 0.41, *p* = 1.000. CA was significantly different in the TD control group (*Mdn* = 14.22) than in both the DS and WS groups, *U* = 30.26, χ^2^ = 4.44, *p* < 0.001, *U* = 27.23, χ^2^ = 5.02, *p* < 0.001, respectively. Because the DS and WS data were normally distributed (i.e., *Z*_Skewness_ < ±2, Kolmogorov–Smirnov, *p* > 0.05), an independent *t*-test was also carried out. It confirmed that the two groups were not significantly different on CA, *t*(30) = 1.38, *p* = 0.178.

Mental age data were normally distributed. A one way ANOVA showed that the groups did not significantly differ on MA, *F*_2,65_ = 0.86, *p* = 0.429, showing that the atypical groups were well matched to one another and to the TD controls.

### Proportion of Target Looking

For each participant and each test trial, the proportion of target looking (PTL) was calculated. PTL is the total amount of time spent looking at the “target” stimulus (i.e., the novel face) as a proportion of the total amount of time spent looking at both the target and the “non-target” stimuli (i.e., the novel face + the familiar face). No data were excluded from these analyses, because none were three standard deviations greater or smaller than the group mean.

**FIGURE 3:**
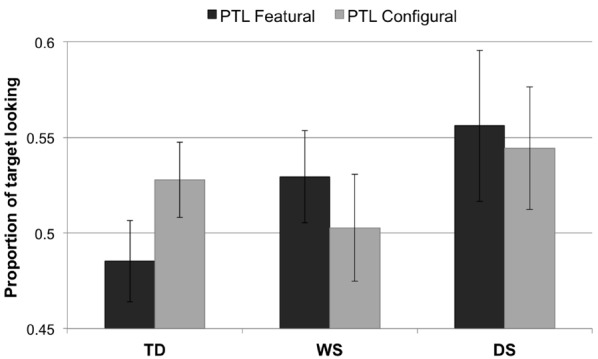
**Proportion of target looking for each condition (featural, configural) and group (TD controls, Williams syndrome, Down syndrome).** A PTL above 0.5 indicates longer looking to the *novel* face than to the familiarized face; a PTL below 0.5 signifies longer looking to the *familiarized* face than to the novel face. Error bars represent ±1 SEM.

The PTL data were normally distributed. There were no main effects of Group, *F*_2,65_ = 1.13, *p* = 0.328, or Condition, *F*_1,65_ = 0.003, *p* = 0.955. Nor was there an interaction effect, *F*_2,65_ = 1.17, *p* = 0.317. Figure 3 illustrates the data from the PTL analysis.

### Longest Look Difference

Proportion of target looking represents an infant’s relative interest over the course of an entire trial/experiment. It is one of the most common measures used by infant researchers to investigate cognitive phenomena. However, it does lack sensitivity. For instance, a participant might look for longer at one face (e.g., the target) and then, after building up an internal representation of it, switch to the other face, simply out of interest, before the trial has ended. This would reduce the likelihood of detecting a difference in looking behavior between the two faces. We therefore used another common, but more sensitive, measure—namely, longest look difference (LLD)—over the first four and last four test trials. We would expect a difference in the first four test trials but not in the last four test trials.

#### Test Trials 1–4

The first four trials (featural left familiar right, configural left familiar right, familiar left featural right, familiar left configural right) were analyzed. As before, data that were three standard deviations greater or smaller than the group mean were excluded from the analyses on a trial basis (data only from two trials from 2 TD participants needed to be excluded). The LLD data were sufficiently normal.

***TD children***

As expected for the first four trials, one-sample *t*-tests indicated that longer looks to the *configurally*-changed face were greater than the chance level of 0 in the TD group, *t*(34) = 2.69, *p* = 0.011, *r* = 0.42. This is considered the normal way for TD participants to process faces. We would, however, also expect TD controls to notice featural changes, albeit less strongly, and indeed a trend emerged with respect to the featurally-changed face, but the analysis did not survive a Bonferroni correction (*p* > 0.05).

***Williams syndrome***

As predicted, a one-sample *t*-test indicated that longer looks to the featurally-changed face were greater than chance in the WS group, *t*(20) = 2.09, *p* = 0.050, *r* = 0.46, but not to the configurally-changed face.

***Down syndrome***

Longer looks were not significantly greater than chance in the DS group (*p* > 0.05) for either the featurally-changed or the configurally-changed faces.

***Intergroup analyses***

**FIGURE 4:**
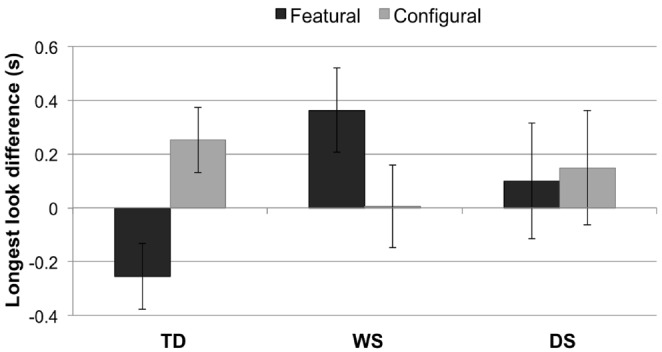
**Longest look difference (single longest look to the novel face minus the single longest look to the familiarized face, in seconds) for each condition (featural, configural) and group (TD controls, Williams syndrome, Down syndrome).** A positive LLD indicates a longer longest look to the *novel* face than to the familiarized face; a negative LLD signifies a longer longest look to the *familiarized* face than to the novel face. Error bars represent ±1 SEM.

A 3 × 2 mixed-design ANOVA with LLD_First_ (featural, configural) as a within-subjects factor and Group (TD control, WS, DS) as a between-subjects factor revealed no main effect of LLD, *F*_1,63_ = 0.25, *p* = 0.617, or Group, *F*_2,63_ = 0.88, *p* = 0.420. In other words, LLD _featural_ did not differ from LLD _configural_, and the three groups did not differ on “LLD.” However, there was an interaction between LLD (featural, configural) and Group, *F*_2,63_ = 5.30, *p* = 0.007, ${\text{η}}_{p}^{2}$ = 0.14 (Figure 4). *Post hoc t*-tests revealed a significant difference between the WS and TD control groups on LLD_ Featural_, *t*(54) = 3.05, *p* = 0.004, *r* = 0.38. No other result survived the Bonferroni correction.

***Order effects***

To investigate order effects, we examined whether LLD for the first presentation of the configurally-changed face was significantly different from LLD for the second presentation of the configurally-changed face. If there were order effects, then we would expect LLD to change between the first and second presentations. Yet no significant changes were detected in the TD control group, *t*(36) = 1.48, *p* = 0.149, WS group, *t*(20) = 1.45, *p* = 0.162, or DS group, *t*(10) = 0.39, *p* = 0.707.

We also compared LLD for the first presentation of the featurally-changed face with LLD for the second presentation of the featurally-changed face. Again, if there were order effects, then we would expect LLD to change between the first and second presentations. Yet no significant changes were detected in the TD control group, *t*(36) = 0.23, *p* = 0.820, WS group, *t*(20) = 0.11, *p* = 0.914, or DS group, *t*(10) = 0.90, *p* = 0.387.

#### Test Trials 5–8

The last four trials were also analyzed. Data that were three standard deviations greater or smaller than the group mean were excluded from the analyses on a trial basis (data only from four trials from 1 TD, 1 WS, and 1 DS participants were excluded). The LLD data were sufficiently normal.

As predicted for the last four trials, neither LLD_ featural_ nor LLD_ configural_ differed from chance in any of the groups (all *p* > 0.05). A 3 × 2 mixed-design ANOVA with LLD _Last_ (featural, configural) as a within-subjects factor and Group (TD control, WS, DS) as a between-subjects factor revealed no main effect of LLD, *F*_1,62_ < 0.01, *p* = 0.968, or Group, *F*_2,62_ = 0.93, *p* = 0.400; nor was there an interaction effect, *F*_2,62_ = 0.42, *p* = 0.662.

## Discussion

Typical face identification entails processing (1) the *features* of a face, (2) the *configuration* of these features or precise variations in the spacing between these features, and (3) the face *holistically* (i.e., as a gestalt). The latter (i.e., holistic face processing) develops in the first months of life in typical development and is relatively proficient in individuals with WS. This has led to claims in the literature that WS (which is characterized by an uneven cognitive profile) presents a unique case of “impaired” and “spared” cognitive modules—with face processing being an example of a spared cognitive module. However, although holistic face processing is proficient in this population, there is evidence that featural and/or configural face processing may be atypical in older children, adolescents, and adults with this syndrome. Indeed, our study revealed that this is the case in infants/toddlers with WS. As predicted, the TD controls showed a significant discrimination between the familiarized and configurally-changed faces, and a weaker discrimination between familiarized and featurally-changed faces. By contrast, and in accordance with our hypothesis, we found that the infants/toddlers with WS failed to discriminate between the faces in the Configural condition, yet showed a novelty preference for the featurally-changed face. This suggests that infants, like older children and adults with WS, have atypical face processing strategies and use predominantly featural rather than configural information to process upright faces.

In other words, although individuals with WS can process faces, our data reveal that they use an atypical strategy to do so. This is an important finding because it means that theorists can no longer argue for the existence of an “intact,” “spared,” or “preserved” face-processing module in WS.

Could theorists argue that face processing is “spared” in infancy and any failure in older children and adults is merely the outcome of a common developmental process that is operating under different (atypical) constraints? This is unlikely, because the present study demonstrates that both featural and configural face processing atypicalities are already evident in infancy. Thus, our data suggest that face processing in WS is already atypical in infancy.

This is a novel finding. It was once thought that face processing was intact in WS. However, evidence has been mounting that one aspect of face processing (configural) develops atypically in older children, adolescents, and adults with WS. Furthermore, a preliminary study hinted that young adults with WS succeed on face recognition tasks by focusing on the features of a face (Karmiloff-Smith, 1997). By manipulating the features of face stimuli and the configuration of these features, our data are the first to confirm that featural face processing is indeed atypical in this population, and that both featural and configural atypicalities are present early in childhood and are thus not the outcome of a protracted developmental process.

As far as concerns infants/toddlers with DS, although they tended to look longer at the novel face in both the Featural and Configural conditions, this difference did not reach significance. It is possible that for DS infants the sample size was too small to detect significant differences. It is also possible that the infants/toddlers with DS may have required a greater number of familiarization trials (than TD infants or those with WS) for them to detect changes in the stimuli. As far as the authors are aware, this is the first study to investigate face processing in such a young population of children with DS, so information on the required number of familiarization trials was not available. Nonetheless, the fact that discrimination is more challenging for infants with DS is a novel finding.

Although our TD infants demonstrated differential looking in the Configural condition (as expected), it is unclear why they showed a trend toward the familiarized face in the Featural condition and a significant bias toward the novel face in the Configural condition. Although children at this young age often demonstrate a bias toward familiarized faces (a *familiarity preference*), infant preferences can be driven by both novelty and familiarity (Fantz, 1964; Zajonc, 1968; Berlyne, 1970; Slater et al., 1983; Bornstein, 1989; Fang et al., 2007). It is possible that the TD infants found the Configural condition easier than the Featural condition; hence a novelty preference was elicited in the former but not in the latter.

Alternatively, it is possible that the TD controls showed a trending familiarity preference for the featurally-changed faces because only local details (the eyes) had changed. Variability in people’s eyes is something with which they already have experience. By contrast, the novelty preference to configurally-changed faces may have arisen because “squashed” and “stretched” faces were extremely novel to them. We hypothesize that the configural-changes were so unexpected that they attracted the TD infants’ longer attention more than changing the shape of the eyes. This hypothesis fits with theories from the face-processing literature: it has been hypothesized that the more discrepant a stimulus is from the observer’s state of knowledge (i.e., from their internal template of face stimuli), the more novel it is to the observer and the more likely it is to elicit a novelty preference (Dember and Earl, 1957; Berlyne, 1960; McCall and McGhee, 1977). In other words, if something is completely new and unknown, it attracts a relatively high level of attention. This would explain why a novelty preference emerged in the Configural condition and a trending familiarity preference was demonstrated in the Featural condition in TD controls.

Whatever the mechanism turns out to be, it is important to note that the TD controls were more sensitive to the configural changes than the featural changes. Furthermore, when we compare the findings from the TD controls with those from the WS group, it suggests that infants/toddlers with WS not only fail to notice configural changes but also that they process featural information atypically. This is because, unlike the controls, the participants with WS showed a novelty preference to the featurally-changed face. In other words, both featural and configural processing of faces is atypical already in infancy in WS.

There are several potential limitations to the study, which will be tested in future research. As mentioned, in this infant study we opted for schematic rather than real faces for several important reasons. First, it has already been shown that infants’ gaze behaviors to naturalistic faces do not differ from their behaviors to schematic faces (e.g., Farroni et al., 2005). For instance, Farroni et al. (2005) found that infants look longer at upright faces than at inverted faces, as a function of contrast polarity *irrespective of whether the face stimuli were schematic or naturalistic*. Second, several studies have shown that the mechanisms involved in processing schematic faces are the same as those involved in processing naturalistic faces (see Johnson et al., 2015, for review). Our choice of schematic faces allowed us to control and manipulate their size and color contrasts, to make them as attractive as possible to infant participants. Additionally, although familiarization paradigms are frequently used in infancy research, one might have preferred a habituation paradigm allowing each infant to find her/his own time to fully encode the model face. However, habituation studies are more prone to subject loss than familiarization studies, and we were dealing with a rare syndrome where subject loss is critical. Moreover, as mentioned, since we used the same familiarized face throughout, all infants had ample time to encode the model face. Thus we opted for a familiarization study because of the rarity of WS and the difficulty in recruiting sufficient numbers of young infants. To our knowledge, this is indeed the first study to examine face processing in neurodevelopmental disorders at such a young age. Yet, to address fundamental questions in psychological theorizing in general, and in face processing in particular, it is crucial to trace developmental trajectories back to their origins in infancy.

Although further research is necessary, our study provides the first evidence that face processing atypicalities are already present very early in the developmental trajectory of individuals with WS. In other words, despite showing subsequent proficiency on standardized face processing tasks, infants/toddlers with WS do not process faces like TD young children. We have also demonstrated that while face processing is atypical in another neurodevelopmental disorder, DS, the two syndromes differ in their strategies and thus the findings with WS cannot be simply explained by low intelligence. In particular, our study highlights the importance of tracing socio-cognitive deficits from very early in development. Finally, our findings indicate that theorists can no longer use the case of WS to support claims that evolution has endowed the human brain with an independent face-processing module.

### Conflict of Interest Statement

The authors declare that the research was conducted in the absence of any commercial or financial relationships that could be construed as a potential conflict of interest.

## References

[B1] Annaz D. Karmiloff-Smith A. Johnson M. H. Thomas M. S. (2009). A cross-syndrome study of the development of holistic face recognition in children with autism, Down syndrome, and Williams syndrome. J. Exp. Child Psychol. 102, 456–486. 10.1016/j.jecp.2008.11.005 19193384

[B2] Bayley N. (1993). Bayley Scales of Infant Development: Manual. San Antonio, TX: The Psychological Corporation.

[B3] Bellugi U. Lichtenberger L. Jones W. Lai Z. St George M. (2000). I. The neurocognitive profile of Williams syndrome: a complex pattern of strengths and weaknesses. J. Cogn. Neurosci. 12 (Suppl. 1), 7–29. 10.1162/089892900561959 10953231

[B4] Bellugi U. Lichtenberger L. Mills D. Galaburda A. Korenberg J. R. (1999). Bridging cognition, the brain and molecular genetics: evidence from Williams syndrome. Trends Neurosci. 22, 197–207. 10.1016/S0166-2236(99)01397-1 10322491

[B5] Bellugi U. Marks S. Bihrle A. Sabo H. (1988a). “Dissociation between language and cognitive functions in Williams syndrome,” in Language Development in Exceptional Circumstances, eds Bishop D. Mogford K. (London: Churchill Livingstone), 177–189.

[B6] Bellugi U. Sabo H. Vaid J. (1988b). “Spatial deficits in children with Williams syndrome,” in Spatial Cognition: Brain Bases and Development, eds Stiles-Davis J. Kritchevsky M. (Hillsdale, NJ: Lawrence Erlbaum Associates, Inc), 273–298.

[B7] Bellugi U. Wang P. P. Jernigan T. L. (1994). “Williams syndrome: an unusual neuropsychological profile,” in Atypical Cognitive Deficits in Developmental Disorders: Implications for Brain Function, eds Broman S. H. Grafman J. (Hillsdale, NJ: Lawrence Erlbaum Associates, Inc), 23–56.

[B8] Benton A. Hamsher K. Varney N. R. Spreen O. (1983). Benton Test of Facial Recognition. Oxford: Oxford University Press.

[B9] Berlyne D. E. (1960). Conflict, Arousal, and Curiosity. New York, NY: McGraw-Hill.

[B10] Berlyne D. E. (1970). Novelty, complexity, and hedonic value. Percept. Psychophys. 8, 279–286. 10.3758/BF03212593

[B11] Bihrle A. M. Bellugi U. Delis D. Marks S. (1989). Seeing either the forest or the trees: dissociation in visuospatial processing. Brain Cogn. 11, 37–49. 10.1016/0278-2626(89)90003-1 2528973

[B12] Bodamer J. (1990). Paper on prosopagnosia. Trans. H. D. Ellis and M. Florence. Cogn. Neuropsychol. 7, 81–105. (Original work published 1947).

[B13] Bornstein R. F. (1989). Exposure and affect: overview and meta-analysis of research, 1968–1987. Psychol. Bull. 106, 265 10.1037/0033-2909.106.2.265

[B14] Busigny T. Graf M. Mayer E. Rossion B. (2010). Acquired prosopagnosia as a face-specific disorder: ruling out the general visual similarity account. Neuropsychologia 48, 2051–2067. 10.1016/j.neuropsychologia.2010.03.026 20362595

[B15] Carey S. Diamond R. (1977). From piecemeal to configurational processing of faces. Science 195, 312–314. 10.1126/science.831281 831281

[B16] Dember W. N. Earl R. W. (1957). Analysis of exploratory, manipulatory, and curiosity behaviors. Psychol. Rev. 64, 91. 10.1037/h0046861 13420283

[B17] Deruelle C. Mancini J. Livet M. O. Casse-Perrot C. De Schonen S. (1999). Configural and local processing of faces in children with Williams syndrome. Brain Cogn. 41, 276–298. 10.1006/brcg.1999.1127 10585239

[B18] Deruelle C. Rondan C. Mancini J. Livet M. (2003). Exploring face processing in Williams syndrome. Cogn. Brain Behav. 7, 157–171.

[B19] Dimitriou D. Leonard H. C. Karmiloff-Smith A. Johnson M. H. Thomas M. S. (2014). Atypical development of configural face recognition in children with autism, Down syndrome and Williams syndrome. J Intellect. Disabil. Res. 59, 422–438. 10.1111/jir.12141 25059077

[B20] Donnai D. Karmiloff-Smith A. (2000). Williams syndrome: from genotype through to the cognitive phenotype. Am. J. Med. Genet. 972, 164–171. 10.1002/1096-8628(200022)97:2<164::AID-AJMG8>3.0.CO;2-F 11180224

[B21] D’Souza D. Booth R. Connolly M. Happé F. Karmiloff-Smith A. (2015). Rethinking the concepts of “local or global processors”: evidence from Williams syndrome, Down syndrome, and Autism Spectrum Disorders. Dev. Sci. 10.1111/desc.12312 [Epub ahead of print]. 26010432 PMC4789488

[B22] D’Souza D. Karmiloff-Smith A. (2011). When modularization fails to occur: a developmental perspective. Cogn. Neuropsychol. 28, 276–287. 10.1080/02643294.2011.614939 22185238

[B23] Duchaine B. Nakayama K. (2005). Dissociations of face and object recognition in developmental prosopagnosia. J. Cogn. Neurosci. 17, 249–261. 10.1162/0898929053124857 15811237

[B24] Duchaine B. C. Nakayama K. (2004). Developmental prosopagnosia and the Benton Facial Recognition test. Neurology 62, 1219–1220. 10.1212/01.WNL.0000118297.03161.B3 15079032

[B25] Fang X. Singh S. Ahluwalia R. (2007). An examination of different explanations for the mere exposure effect. J. Consum. Res. 34, 97–103. 10.1086/513050

[B26] Fantz R. L. (1964). Visual experience in infants: decreased attention to familiar patterns relative to novel ones. Science 146, 668–670. 10.1126/science.146.3644.668 14191712

[B27] Farran E. K. Karmiloff-Smith A. (2012). Neurodevelopmental Disorders Across the Lifespan: A Neuroconstructivist Approach. Oxford: Oxford University Press.

[B28] Farroni T. Johnson M. H. Menon E. Zulian L. Faraguna D. Csibra G. (2005). Newborns’ preference for face-relevant stimuli: efforts of contrast polarity. Proc. Natl. Acad. Sci. U.S.A. 102, 17245–17250. 10.1073/pnas.0502205102 16284255 PMC1287965

[B29] Golarai G. Hong S. Haas B. W. Galaburda A. M. Mills D. L. Bellugi U. (2010). The fusiform face area is enlarged in Williams syndrome. J. Neurosci. 30, 6700–6712. 10.1523/jneurosci.4268-09.2010 20463232 PMC3670816

[B30] Goren C. C. Sarty M. Wu P. Y. (1975). Visual following and pattern discrimination of face-like stimuli by newborn infants. Pediatrics 56, 544–549. 1165958

[B31] Grice S. J. de Haan M. Halit H. Johnson M. H. Csibra G. Grant J. (2003). ERP abnormalities of illusory contour perception in Williams syndrome. Neuroreport 14, 1773–1777. 14534418 10.1097/00001756-200310060-00003

[B32] Hollich G. (2005). Supercoder: A Program for Coding Preferential Looking (version 1.5). West Lafayette, IN: Purdue University.

[B33] Johnson M. H. Dziurawiec S. Ellis H. Morton J. (1991). Newborns’ preferential tracking of face-like stimuli and its subsequent decline. Cognition 40, 1–19. 10.1016/0010-0277(91)90045-6 1786670

[B34] Johnson M. H. Senju A. Tomalski P. (2015). The two-process theory of face processing: modifications based on two decades of data from infants and adults. Neurosci. Biobehav. Rev. 50, 169–179. 10.1016/j.neubiorev.2014.10.009 25454353

[B35] Kanwisher N. (2000). Domain specificity in face perception. Nat. Neurosci. 3, 759–763. 10.1038/77664 10903567

[B36] Kanwisher N. (2010). Functional specificity in the human brain: a window into the functional architecture of the mind. Proc. Natl. Acad. Sci. U.S.A. 107, 11163–11170. 10.1073/pnas.1005062107 20484679 PMC2895137

[B37] Kanwisher N. McDermott J. Chun M. M. (1997). The fusiform face area: a module in human extrastriate cortex specialized for face perception. J. Neurosci. 17, 4302–4311. 9151747 10.1523/JNEUROSCI.17-11-04302.1997 PMC6573547

[B38] Kanwisher N. Stanley D. Harris A. (1999). The fusiform face area is selective for faces not animals. Neuroreport 10, 183–187. 10.1097/00001756-199901180-00035 10094159

[B39] Karmiloff-Smith A. (1997). Crucial differences between developmental cognitive neuroscience and adult neuropsychology. Dev. Neuropsychol. 13, 513–524. 10.1080/87565649709540693

[B40] Karmiloff-Smith A. (1998). Development itself is the key to understanding developmental disorders. Trends Cogn. Sci. 2, 389–398. 10.1016/S1364-6613(98)01230-3 21227254

[B41] Karmiloff-Smith A. D’Souza D. Dekker T. M. Van Herwegen J. Xu F. Rodic M. (2012). Genetic and environmental vulnerabilities in children with neurodevelopmental disorders. Proc. Natl. Acad. Sci. U.S.A. 109, 2, 17261–17265. 10.1073/pnas.1121087109 23045661 PMC3477396

[B42] Karmiloff-Smith A. Thomas M. Annaz D. Humphreys K. Ewing S. Brace N. (2004). Exploring the Williams syndrome face-processing debate: the importance of building developmental trajectories. J. Child Psychol. Psychiatry 45, 1258–1274. 10.1111/j.1469-7610.2004.00322.x 15335346

[B43] Laing E. Butterworth G. Ansari D. Gsödl M. Longhi E. Panagiotaki G. (2002). Atypical development of language and social communication in toddlers with Williams syndrome. Dev. Sci. 5, 233–246. 10.1111/1467-7687.00225

[B44] Leder H. Bruce V. (2000). When inverted faces are recognized: the role of configural information in face recognition. Q. J. Exp. Psychol. A 53, 513–536. 10.1080/713755889 10881616

[B45] Liu S. Anzures G. Ge L. Quinn P. C. Pascalis O. Slater A. M. (2013). Development of recognition of face parts from unfamiliar faces. Infant Child Dev. 22, 165–179. 10.1002/icd.1781 24009474 PMC3760427

[B46] Maurer D. Grand R. L. Mondloch C. J. (2002). The many faces of configural processing. Trends Cogn. Sci. 6, 255–260. 10.1016/S1364-6613(02)01903-4 12039607

[B47] McCall R. B. McGhee P. E. (1977). “The discrepancy hypothesis of attention and affect in infants,” in The Structuring of Experience, eds Uzgiris I. C. Weizmann F. (New York, NY: Plenum), 179–210.

[B48] McCarthy G. Puce A. Gore J. C. Allison T. (1997). Face-specific processing in the human fusiform gyrus. J. Cogn. Neurosci. 9, 605–610. 10.1162/jocn.1997.9.5.605 23965119

[B49] Mervis C. B. Bertrand J. (1997). “Developmental relations between cognition and language: evidence from Williams syndrome,” in Research on Communication and Language Disorders: Contributions to Theories of Language Development, eds Adamson L. B. Romski M. A. (New York, NY: Brookes), 75–106.

[B50] Mills D. Alvarez T. D. St. George M. Appelbaum L. G. Bellugi U. Neville H. (2000). III. Electrophysiological studies of face processing in Williams syndrome. J. Cogn. Neurosci. 12, 47–64. 10.1162/089892900561977 10953233

[B51] Mobbs D. Garrett A. S. Menon V. Rose F. E. Bellugi U. Reiss A. L. (2004). Anomalous brain activation during face and gaze processing in Williams syndrome. Neurology 62, 2070–2076. 10.1212/01.WNL.0000129536.95274.DC 15184616

[B52] Mondloch C. J. Le Grand R. Maurer D. (2002). Configural face processing develops more slowly than featural face processing. Perception 31, 553–566. 10.1068/p3339 12044096

[B53] Moscovitch M. Winocur G. Behrmann M. (1997). What is special about face recognition? Nineteen experiments on a person with visual object agnosia and dyslexia but normal face recognition. J. Cogn. Neurosci. 9, 555–604. 10.1162/jocn.1997.9.5.555 23965118

[B54] Pani J. R. Mervis C. B. Robinson B. F. (1999). Global spatial organization by individuals with Williams syndrome. Psychol. Sci. 10, 453–458. 10.1111/1467-9280.00186

[B55] Piepers D. W. Robbins R. A. (2012). A review and clarification of the terms “holistic,” “configural,” and “relational” in the face perception literature. Front. Psychol. 3:559. 10.3389/fpsyg.2012.00559 23413184 PMC3571734

[B56] Puce A. Allison T. Asgari M. Gore J. C. McCarthy G. (1996). Differential sensitivity of human visual cortex to faces, letter-strings, and textures: a functional magnetic resonance imaging study. J. Neurosci. 16, 5205–5215. 8756449 10.1523/JNEUROSCI.16-16-05205.1996 PMC6579313

[B57] Rossen M. L. Jones W. Wang P. P. Klima E. S. (1995). Face processing: remarkable sparing in Williams syndrome. J. Genet. Couns. 6, 138–140.

[B58] Slater A. Morison V. Rose D. (1983). Locus of habituation in the human newborn. Perception 12, 593–598. 10.1068/p120593 6676710

[B59] Tager-Flusberg H. Plesa-Skwerer D. Faja S. Joseph R. M. (2003). People with Williams syndrome process faces holistically. Cognition 89, 11–24. 10.1016/S0010-0277(03)00049-0 12893122

[B60] Tanaka J. W. Farah M. J. (1993). Parts and wholes in face recognition. Q. J. Exp. Psychol. A 46, 225–245. 10.1080/14640749308401045 8316637

[B61] Tong F. Nakayama K. Moscovitch M. Weinrib O. Kanwisher N. (2000). Response properties of the human fusiform face area. Cogn. Neuropsychol. 17, 257–280. 10.1080/026432900380607 20945183

[B62] Turati C. Di Giorgio E. Bardi L. Simion F. (2010). Holistic face processing in newborns, 3-month-old infants, and adults: evidence from the composite face effect. Child Dev. 81, 1894–1905. 10.1111/j.1467-8624.2010.01520.x 21077871

[B63] Wang P. P. Doherty S. Rourke S. B. Bellugi U. (1995). Unique profile of visuo-perceptual skills in a genetic syndrome. Brain Cogn. 29, 54–65. 10.1006/brcg.1995.1267 8845123

[B64] Wilson B. Cockburn J. Baddeley A. D. (1986). The Rivermead Behavioural Memory Test. Bury St Edmunds: Thames Valley Test Company.

[B65] Yin R. K. (1969). Looking at upside-down faces. J. Exp. Psychol. 81, 141–145. 10.1037/h0027474

[B66] Young A. W. Hellawell D. Hay D. C. (1987). Configurational information in face perception. Perception 16, 747–759. 10.1068/p160747 3454432

[B67] Zajonc R. B. (1968). Attitudinal effects of mere exposure. J. Pers. Soc. Psychol. 9, 1–27. 10.1037/h00258485667435

